# Reuse of face masks among adults in Hong Kong during the COVID-19 pandemic

**DOI:** 10.1186/s12889-021-11346-y

**Published:** 2021-06-29

**Authors:** Linda Yin-king Lee, Issac Chun-wing Chan, Owen Pak-man Wong, Yaki Hoi-ying Ng, Crystal Kit-ying Ng, Max Hin-wa Chan, Joe Ka-chun Ng, Hailey Hei-tung Koo, Suk-ting Lam, Ada Cho-wai Chu, Rachel Yuen-shan Wong, Heidi Po-ying Leung, Angel Lok-ching Pun

**Affiliations:** School of Nursing and Health Studies, The Open University of Hong Kong, Ho Man Tin, Kowloon, Hong Kong

**Keywords:** Face mask, COVID-19, Hong Kong

## Abstract

**Background:**

During the COVID-19 pandemic, over 99% of adults in Hong Kong use face masks in public. With the limited supply of face masks in the market and the uncertainty about the future development of COVID-19, reusing face masks is a legitimate way to reduce usage. Although this practice is not recommended, reusing face masks is common in Hong Kong. This study aimed to examine the practice of reusing face masks among adults in Hong Kong during the COVID-19 pandemic and its association with their health beliefs toward this health crisis.

**Methods:**

A cross-sectional descriptive study was conducted. A quota sample of 1000 adults was recruited in Hong Kong in April 2020. Guided by the Health Belief Model, the subjects were invited to answer questions on their practice of reusing face masks and health beliefs toward COVID-19 through telephone interview. Their practice on reuse, storage, and decontamination of used face masks were summarized by descriptive statistics. The difference in health beliefs between the subjects who reused and did not reuse face masks was examined by conducting an independent t test. The association between health beliefs and reuse of face masks was determined by conducting a logistic regression analysis.

**Results:**

One-third (*n* = 345, 35.4%) of the subjects reused face masks in an average of 2.5 days. Among them, 207 subjects stored and 115 subjects decontaminated their used face masks by using various methods. The subjects who reused face masks significantly perceived having inadequate face masks (*t* = 3.905; *p* <  0.001). Having a higher level of perception of having inadequate face masks increased the likelihood of reusing face masks (OR = 0.784; CI 95%: 0.659–0.934; *p* = 0.006).

**Conclusion:**

Despite having 90 face masks in stock, the adults who reused face masks significantly perceived that they had inadequate face masks. Concerted effort of health care professionals, community organizations, and the government will improve individuals’ practice in use of face masks and alleviate their actual and perceived feeling of having inadequate face masks, which lead them to reuse.

## Background

The COVID-19 pandemic outbreak originated in Wuhan City, which is the capital of Hubei Province, China in early January 2020 [1]. It spread across China and to many countries and regions rapidly. National and international bodies have escalated their disease response level to address the continuous pandemic outbreak. The Hong Kong Government activated the Emergency Response Level, which was the highest emergency level, under the “Preparedness and Response Plan for Novel Infectious Disease of Public Health Significance (2020)” on January 25, 2020 [2]. The World Health Organization (WHO) increased the assessment of the risks of spread and influence of COVID-19 to the global level on February 28, 2020 [3] and classified the disease as a controllable pandemic on March 12, 2020 [4]. Within two months, COVID-19 became an international health matter that concerned most people in the world.

For self-protection and to contain the spread of COVID-19 in public, experts and health authorities recommend individuals to wear face masks [5, 6]. When this mask is used in a non-clinical context, it is commonly called a face mask. When this mask is used in a clinical context, it is named as a surgical mask. A face mask is a loose-fitting non-pharmaceutical device designed to cover the nose, mouth, and chin. It provides a physical barrier against respiratory infectious droplets [7]. It is designed for single-use only and should be disposed immediately after use [8]. The use of face masks for public health rationale is particularly essential in the current COVID-19 pandemic because this disease can be spread even by asymptomatic individuals and has widely spread in communities already [9, 10]. A systematic review and meta-analysis support the effectiveness of using face masks in reducing the spread of COVID-19 infection [11]. At the national level, countries and regions, such as Mainland China, Hong Kong, Macau, Japan, and South Korea, have adopted mass use of face masks as their public health policy. At the individual level, people in Hong Kong have responded quickly as soon as the first case of COVID-19 was confirmed on January 23, 2020. Population surveys in Hong Kong indicated that the percentage of respondents who wore face masks in public increased significantly within 3 months from 74.5% in January 2020 to 98.8% in March 2020 [12].

However, the pandemic nature of COVID-19 causes shortages of face masks worldwide [13, 14]. Although face masks are intended for single-use only, reusing them in public is a common practice [15]. The practice of reusing face masks represents a public health issue that deserves immediate attention and scientific investigation.

### Reusing face masks: prevalence

In Brazil, 55.8% of adult face mask users reuse face masks, among which 27.2% reuse a face mask for one to two times [16]. In Hong Kong, 54% of adult face mask users reuse face masks, among which 83.3% reuse a single face mask for one to two times [15]. In another study, 17.2% of older adults reuse a face mask for more than two times [17]. Comparison of findings between countries/regions should be done with caution because of the differences in public health policy and practice. In Brazil, the Ministry of Health advises its population to wear sanitized homemade masks to overcome the shortage of face masks. As much as 77.7% of the adults in Brazil use fabric/cotton homemade masks exclusively or in combination with other types of mask, such as surgical masks, N95 masks, and paper or gauze masks. Only 27.8% of the adults use face masks exclusively [16]. In Hong Kong, however, homemade masks are not recommended. As much as 98.8% of the Hong Kong population use face masks [12]. Moreover, the research methods of previous studies should be considered when interpreting their findings. Some online surveys adopted in previous studies have excluded digital illiterates who might be less financially resourceful and of lower education level [15–17]. Furthermore, convenience sampling has been adopted, which might have induced selection bias [17]. Thus, the representativeness of their samples might have been reduced.

### Reusing face masks: storage and decontamination

The reuse of a face mask refers to the practice of using the same face mask repeatedly with the act of removing it after each use and putting it on again for the next use. Between the act of removing and putting on the used face mask again, people commonly handle the used face mask using two distinctive procedures, namely, storing and decontaminating the used face mask. This definition is developed from those definitions for the reuse of N95 respirators that are made by the literature and international health care bodies [18–20].

People commonly store their used face masks in paper bags or plastic bags. The availability of specially designed plastic folders and boxes in the market facilitates people to store used face masks and reinforces their belief that this practice is appropriate. Once a face mask is used, its outer surface is contaminated. If a used face mask is not stored properly, the pathogens from the outer surface can be transferred to the inner surface of the face mask or to the hands and other body parts that have touched the outer surface of the face mask. In any of the above situations, the pathogens can eventually be transferred to the mucous membranes of the users’ face. Thus, users will have a risk of acquiring infection. Although this health risk is mentioned by the international body for the reuse of respirators in health care settings [18], applying this health risk to the reuse of face masks in public setting is still logical and reasonable.

People also decontaminate their used face masks by using alcohol, hot water, steam, ultraviolet light, sunlight, or wind [21]. The availability of faulty information in the Internet further makes people believe that these methods are appropriate. In Hong Kong, some people believe that steaming a face mask can make it reusable. Despite the warning of health experts against this practice, people, especially the poor and old ones, follow the hearsay and steam their face masks [22]. If a used face mask has undergone improper decontamination, undesirable outcomes will arise. If the decontamination method is ineffective, the pathogens that are staying on the face masks cannot be killed. Although the decontamination method is effective, the structural integrity of a used face mask may still be damaged [23]. A technique that can destroy the viruses effectively does not necessarily imply that it can retain the protective blocking structure of a used face mask during decontamination [24]. Anyone who is using an improperly decontaminated face mask may have a risk of not receiving any protection from it.

The decontamination of used face masks is an important procedure to ensure safety in reuse. Thus far, considerable research on the decontamination of used face masks and the maintenance of the blocking efficacy of these devices after decontamination is relevant to the reuse of N95 respirators in a health care setting [23, 25–29].

Existing guidelines also specify the proper ways for the limited reuse of N95 respirators in health care settings [18, 30]. Commonly, the recommended decontamination methods, such as ultraviolet germicidal irradiation, autoclave, gamma irradiation, vaporized hydrogen peroxide, and ethylene oxide, require specific facilities. Moreover, the materials and facilities are neither available in a community setting nor suitable for personal use [23]. Thus, existing evidence cannot be applicable for public use. Discussions on the reuse of face masks in a community setting are even rare.

### Reusing face masks: a health-preventive behavior

The decision to reuse a face mask can be related to various factors. The Health Belief Model specifies that individuals’ likelihood of performing a specific health-preventive behavior is influenced by their perceived susceptibility to and perceived severity of the targeted disease and the weighting of perceived benefits of and perceived barriers to adopt the health-preventive behavior. These perceptions are also influenced interchangeably by individuals’ self-efficacy to perform the heath-preventive behavior, their socio-demographic characteristics, and the presence of cues to action [31]. The Health Belief Model is one of the most widely used models in health behavior research [32]. This model attempts to predict health-preventive behaviors by considering the key constructs of the model. During the COVID-19 pandemic, the Health Belief Model has been used to predict health-preventive behaviors, such as adoption of personal hygiene measures, maintenance of social distancing, and compliance with the recommendations of the government [33, 34]. Moreover, this model has been used to investigate the use and reuse of face masks [15, 17]. Tentatively, a study in older adults revealed that a higher perceived efficacy of practicing preventive measures is associated with the use of face masks, poorer cues to preventive measures are associated with the reuse of face masks, and a stronger belief in disease severity is associated with the use and reuse of face masks [17]. Other than health beliefs, gender and health experience are found to be associated with the reuse of face masks [16].

The reuse of face masks, despite not being a recommended behavior, is surely a health-preventive behavior performed by people to prevent being infected by COVID-19. Accordingly, this study considers the Health Belief Model to be an appropriate tool in explaining the present phenomenon.

## Methods

### Research aim and objectives

Given the above analysis, this study aimed to examine the reuse of face masks among adults in Hong Kong during the COVID-19 pandemic. Specifically, this study had three objectives:

1. To examine the practice of reusing face masks, in terms of storage and decontamination, among adults in Hong Kong.
2. To examine the health beliefs toward COVID-19 and the use of face masks as a health-preventive behavior among adults in Hong Kong.
3. To examine the association between health beliefs and the reuse of face masks among adults in Hong Kong.

Adults constitute the major population in Hong Kong. They have developed their own health beliefs and can decide whether to engage in a health-preventive behavior. Thus, adults represent an appropriate target population for this study. Findings of this study are insightful to ascertain adults’ practice and explain the situation of reusing face masks in Hong Kong during the COVID-19 pandemic. By targeting the various constructs of the Health Belief Model, this study aimed to aid the development of effective interventions to change the public’s health-preventive behaviors.

### Design

This quantitative study adopted a cross-sectional descriptive design.

### Setting and sample

This study was conducted in Hong Kong during the first two weeks of April 2020. In this period, Hong Kong was experiencing the second wave of the COVID-19 outbreak (around March 6, 2020 to April 16, 2020). As of April 1, 2020, Hong Kong reported a total of 737 confirmed cases and 4 deaths [35].

This study recruited a quota sample of 1000 adults. Quota sampling increases the representativeness of the sample when a sampling frame is unavailable and probability sampling method is impossible [36]. Fourteen strata were formed (Table 1). The proportion in these strata was determined by the latest Hong Kong census results, showing the age and gender distribution of the adult proportion in 2016 [37]. Once the size of each stratum was determined, the subjects in each stratum were recruited conveniently through the network of the researchers. Using this method could not avoid recruiting subjects who knew the researchers. However, this method was feasible during a respiratory pandemic outbreak because all types of social activity were reduced to minimal. On the basis of Cochran formula, a sample size of 1000 was adequate to attain a confidence level of 99% and a margin of error of 5% for a population of 6,165,223 Hong Kong adults aged 20 or above.

**Table 1:** Age and gender distribution of Hong Kong population and study sample

Age	Gender	Hong Kong population 2016 (*n* = 6,165,223)		Study sample (*n* = 1000)	
		Frequency	Percentage	Frequency	Percentage
20–29	Male	448,018	7.3	73	7.3
	Female	507,292	8.2	82	8.2
30–39	Male	460,970	7.5	75	7.5
	Female	687,555	11.2	112	11.2
40–49	Male	474,874	7.7	77	7.7
	Female	661,968	10.7	107	10.7
50–59	Male	599,552	9.7	97	9.7
	Female	666,562	10.8	108	10.8
60–69	Male	440,629	7.1	71	7.1
	Female	450,353	7.3	73	7.3
70–79	Male	213,407	3.5	35	3.5
	Female	213,794	3.5	35	3.5
80+	Male	133,116	2.2	22	2.2
	Female	207,133	3.3	33	3.3
Total	Male	2,770,566	44.9	449	44.9
	Female	3,394,657	55.1	551	55.1
	Both genders	6,165,223	100.0	1000	100.0

The inclusion criteria were: (1) being an adult (aged 20 or above); (2) being a Hong Kong resident; (3) uses face mask; (4) can communicate in Cantonese (a common dialect in Hong Kong); and (5) lives in Hong Kong during the COVID-19 pandemic. The exclusion criteria were: (1) lives in a residential care home; or (2) being hospitalized.

### Variable

The variable of interest was the reuse of face mask. It is operationally defined as the practice of using the same face mask repeatedly with the act of removing it after each use and putting it on again for the next use. This definition is modified from those definitions for the reuse of N95 respirators that are made by the literature and international health care bodies [18–20].

Between the act of removing and putting on the used face mask again, people commonly handle the used face mask using two distinctive procedures, namely, storing and decontaminating the used face mask. This interpretation was made by the research team through a number of measures, including observation of citizens’ behaviors, interviews with the citizens, and review of newspapers and television programs.

### Data collection

A questionnaire was developed. It included three parts. Part A provided questions on sociodemographic and health characteristics, number of masks at hand, sources of face masks, and the practice of reusing a face mask (yes/no). If a subject indicated reuse of face masks, he/she was asked to indicate the number of days that a face mask was used (duration of use).

Part B consisted of four questions. One question was about the storage of a used face mask (yes/no), followed by one question on the storage method. Five options were provided. Moreover, one question was about the decontamination of a used face mask (yes/no), followed by one question on the decontamination method. Eight options were provided. The subjects who reused a face mask were asked to choose the applicable option(s). An expert panel, comprising of three academics and three clinical nurses on public health, was formed to evaluate the content validity of the questionnaire. This part of the questionnaire demonstrated high content validity (content validity index = 0.98) [38]. One hundred adults were invited to complete the questionnaire twice at a two-week interval. The test–retest result indicated good test–retest reliability (kappa coefficient = 0.86) [39].

Part C consisted of five questions on health beliefs toward COVID-19 and the use of face masks as a health-preventive behavior. These questions were set in accordance with the literature on Health Belief Model and expert advice. The five questions on health beliefs covered the perceived susceptibility to COVID-19, perceived seriousness of COVID-19, perceived benefit of using face masks, perceived barrier in using face masks, and perceived self-efficacy to protect oneself from COVID-19. Each question was rated on a four-point Likert scale (1 = strongly disagree, 2 = disagree, 3 = agree, 4 = strongly agree). The content validity of the five questions was evaluated by the aforementioned expert panel. The result demonstrated high content validity (content validity index = 0.91) [38]. The two-week test–retest reliability of the five questions was conducted on the abovementioned 100 adults. The result demonstrated good test–retest reliability (intraclass correlation coefficient = 0.83) [39, 40].

The subjects were invited to answer the questions that were read by a researcher through a telephone interview. Using telephone interview was considered as a desirable data collection method during a respiratory pandemic because it could minimize physical contact. It provided a good alternative to face-to-face interview.

### Procedures

On the basis of the predetermined number of subjects in each of the 14 strata and the selection criteria, the researchers identified the potential subjects from their networks and made individual telephone call to them. The researcher read all the questions in the questionnaire according to a standardized protocol, which was prepared beforehand, thereby ensuring consistency in implementation.

### Statistical analyses

Descriptive statistics were performed to describe the subjects’ sociodemographic and health characteristics, practices in reusing face masks, and health beliefs. Chi-square test was used to compare the difference in proportion of the various sociodemographic and health characteristics between subjects who reused and did not reuse face masks. Independent t test was used to compare the difference in health beliefs between subjects who reused and did not reuse face masks. To identify their influence on the reuse of face mask, significant variables in the above univariate analysis were entered into a binary logistic regression model with the reuse of face mask as the dependent variable. Verification was made using odds ratio (OR) and the respective confidence interval (CI) at 95%. The level of significance was set at *p* <  0.05.

### Ethical considerations

The present study involved human participants. It was conducted in accordance with the Declaration of Helsinki. Ethical approval was obtained from the Research Ethics Committee of The Open University of Hong Kong (Ref: HE-SF2020/04). All methods were carried out in accordance with the relevant guidelines and regulations. An invitation letter was prepared. The letter explicitly stated the nature of the research, expected involvement from the subjects, and subjects’ right. The subjects were informed about the voluntary participation and anonymity in data handling. Subjects gave verbal informed consent.

## Results

A total of 1052 potential subjects were approached, and 95% agreed to participate in the study. Finally, 1000 eligible subjects were recruited. No missing data existed. All the data were valid for analysis. The subjects’ age and gender resembled the characteristics of the Hong Kong population. Nearly half of the subjects attained at least tertiary educational level. Majority of the subjects lived with others (89.5%) and considered themselves as healthy (83.5%). On average, each subject had 90 face masks. Assuming that a person uses a face mask in each day, having a stock of 90 face masks is adequate for a three-month use. One-third (*n* = 345, 34.5%) of the subjects reused their face masks. Age, education level, living status, and health status were found to be significantly associated with the reuse of face masks, as shown in Table 2.

**Table 2:** Sociodemographic and health characteristics (*n* = 1000)

Sociodemographic and health characteristic	Frequency (percentage)			*x*^2^	*p*
	All subjects (*n* = 1000)	Subjects who reuse face masks			
		Yes (*n* = 345)	No (*n* = 655)		
Age				82.511	< 0.001***
20–29	155 (15.5)	49 (31.6)	106 (68.4)		
30–39	187 (18.7)	34 (18.2)	153 (81.8)		
40–49	184 (18.4)	48 (26.1)	136 (73.9)		
50–59	205 (20.5)	70 (34.1)	135 (65.9)		
60–69	144 (14.4)	75 (52.1)	69 (47.9)		
70–79	70 (7.0)	30 (42.9)	40 (57.1)		
80+	55 (5.5)	39 (70.9)	16 (29.1)		
Gender				0.251	0.616
Male	450 (45.0)	151 (33.6)	299 (66.4)		
Female	550 (55.0)	194 (35.3)	356 (64.7)		
Education level					
Below primary	32 (3.2)	19 (59.4)	13 (40.6)	54.632	< 0.001***
Primary	102 (10.2)	65 (63.7)	37 (36.3)		
Secondary	392 (39.2)	117 (29.8)	275 (70.2)		
Tertiary and above	474 (47.4)	144 (30.4)	330 (69.6)		
Living status				10.987	0.001**
Live alone	105 (10.5)	52 (49.5)	53 (50.5)		
Live with others	895 (89.5)	293 (32.7)	602 (67.3)		
Health status					
Healthy (not on medication)	835 (83.5)	263 (31.5)	572 (68.5)	19.398	< 0.001***
With chronic diseases (on long-term medication)	165 (16.5)	82 (49.7)	83 (50.3)		
		Mean (SD)		*t*	*p*
Number of face masks at hand	90 (192.7)	83	93	−0.829	0.408

### Use and reuse of face masks

The subjects obtained face masks from various sources, and the most common sources were by oneself (68.0%) and family members (55.9%), as shown in Table 3. Of those 345 subjects who reused face masks, they, on average, reused a face mask for 2.5 days. Among them, 60% stored their used face masks between each time they used the mask. The commonly adopted methods were covering the used face mask by tissue paper (*n* = 198, 57.4%) and placing it inside a plastic bag (*n* = 110, 31.9%) or a paper bag (*n* = 100, 29.0%). The subjects (*n* = 138, 40.0%) who did not store used face masks simply placed the mask in an open area (*n* = 119, 34.5%) or anywhere that was convenient to them (*n* = 19, 5.5%). Among the subjects who reused face masks, one-third (*n* = 115, 32.2%) had a habit to decontaminate their used face masks. Among them, the commonly adopted methods were using wind (*n* = 82, 23.8%), sunlight (*n* = 42, 12.2%), and alcohol (*n* = 40, 11.6%), as shown in Table 4.

**Table 3:** Use and reuse of face masks (*n* = 1000)

Performance	Frequency	Percentage
Source of face masks		
Oneself	680	68.0
Family members	559	55.9
Home storage	408	40.8
Other people	296	29.6
Workplace	258	25.8
Others	8	0.8
Reuse of face masks		
Yes	345	34.5
No	655	65.5

**Table 4:** Duration of use, storage and decontamination of used face masks (*n* = 345)

Performance	Mean	SD
Day of use (per mask)	2.5	5.5
	Frequency	Percentage
Storage of used face masks (practice)		
Yes	207	60.0
No	138	40.0
Storage of used face masks (method)*		
Placing the face mask inside a pocket	85	24.6
Placing the facemask inside a plastic bag	110	31.9
Placing the face mask inside a paper bag	100	29.0
Covering the face mask by tissue paper	198	57.4
Others	9	2.6
Decontamination of used face masks (practice)		
Yes	115	32.2
No	242	67.8
Decontamination of used face masks (method)**		
Using alcohol	40	11.6
Using detergent	9	2.6
Using sunlight	42	12.2
Using UV light	11	3.2
Using steam	8	2.3
Using iron heat	5	1.4
Using wind	82	23.8
Others	3	0.9

* Among the 207 subjects who stored the used face masks, some subjects adopted more than one method to store the used face masks

** Among the 115 subjects who decontaminated the used face masks, some subjects adopted more than one method to decontaminate the used face masks

### Health beliefs

Regarding their health beliefs toward COVID-19, the subjects tended to be neutral about their susceptibility to COVID-19 (perceived susceptibility, mean = 2.2). However, they agreed that COVID-19 was a serious disease (perceived seriousness, mean = 3.4). Regarding the health belief on using face masks as a health-preventive behavior, the subjects agreed that wearing a face mask could protect them from acquiring COVID-19 (perceived benefit, mean = 3.2). However, they tended to be neutral on whether they had inadequate face masks (perceived barrier, mean = 2.3) and the ability to protect themselves from acquiring the disease (perceived self-efficacy, mean = 2.7). Although the overall sample did not believe that they had inadequate face masks, a comparison between the subjects who reused and did not reuse their face masks revealed that the former significantly perceived that they had inadequate face masks (*t* = 3.905, *p* <  0.001), as shown in Table 5.

**Table 5:** Health beliefs and reuse of face masks (*n* = 1000)

Health belief	Mean (SD)			*t*	*p*
	All subjects (*n* = 1000)	Subjects who reuse face masks			
		Yes (*n* = 345)	No (*n* = 655)		
Perceived susceptibility: I am susceptible to COVID-19	2.2 (0.8)	2.1 (0.8)	2.2 (0.8)	−1.426	0.154
Perceived seriousness: COVID-19 is a serious disease	3.4 (0.7)	3.4 (0.7)	3.4 (0.6)	0.806	0.420
Perceived benefit: Wearing a face mask can protect me from acquiring COVID-19	3.2 (0.6)	3.2 (0.6)	3.2 (0.6)	−0.081	0.936
Perceived barrier: I have inadequate face masks	2.3 (0.8)	2.5 (0.9)	2.2 (0.7)	3.905	< 0.001***
Perceived self-efficacy: I am able to protect myself form acquiring COVID-19	2.7 (0.7)	2.7 (0.8)	2.8 (0.7)	−1.140	0.255

The effects of other significant factors in the univariate analysis were controlled. Accordingly, a binary logistic regression analysis revealed that perceived having inadequate face masks was a significant factor associated with the reuse of face masks (OR = 0.784; CI 95%: 0.659–0.934; *p* = 0.006). The higher the level of perceived having inadequate face masks was, the higher the likelihood of reusing face masks would be.

## Discussion

When the COVID-19 pandemic started in early 2020, Hong Kong people acted swiftly and collectively. A previous study indicated that 98.8% of the Hong Kong population used face masks [12], and the present study reported that 34.5% of the subjects had a habit of reusing face masks. Furthermore, it described the practice of storing and decontaminating the used face masks, which have not been examined in the literature. Despite being a common practice, reusing face masks can impose a health risk to the users and is not recommended. The findings highlight a significant public health issue that requires attention.

### Prolonged use and reuse, improper storage, and decontamination of face masks

The subjects who reused face masks reported using the same face mask for 2.5 days. Commonly, manufacturers do not recommend using the same face mask longer than a day. In prolonged use, the secretions from nose and mouth coat the inner surface of the face masks. This coating eventually turns into a breeding ground for pathogens, thereby increasing the health risk of users and creating a route of transfer of the pathogens to the other people. However, people commonly overlook this health risk because used face masks can appear clean in naked eyes.

Among the subjects who reused face masks, 60.0% had a habit of storing a used face mask. The act of storing is believed to protect face masks from being contaminated by the external environment while it is not in use. Without any scientific evidence to support the appropriateness of using those storage methods that were adopted by the subjects, claiming that used face masks can be safely protected during storage is difficult. Moreover, improper handling during the storage procedures increases the chance of transferring the pathogens from the surface of a used face mask to the user’s body and may eventually cause infection.

Among the subjects who reused face masks, 32.2% had a habit of decontaminating used face masks. The act of decontamination is believed to minimize potential infection risk and enhance safety in reuse [23]. However, it should be done properly. Improper decontamination may either damage the blocking structure of the face masks or be unable to inactivate the pathogens completely [21]. In comparison with those established methods and well-delineated procedures that are used to decontaminate N95 respirators in hospitals [23, 26], all the decontamination methods that were adopted by the subjects in this study are informal. Furthermore, no standard guideline specifies how the decontamination procedures should be done in household or personal settings. Individuals may adopt different practices. With this background, we could not claim that the subjects had decontaminated their used face masks properly.

Amid the COVID-19 pandemic, countries and regions worldwide experience shortage of face masks. People are finding ways to make each face mask last longer. Scientists in Hong Kong are studying the option of reusing face masks [41]. Before the establishment of any scientific evidence and guidelines, reusing face masks remains to be a high-risk practice.

### Perceived susceptibility to and perceived seriousness of COVID-19

Different from the high levels of perceived susceptibility to COVID-19, as reported during the early onset of disease [42], the subjects in this study conducted in April 2020 tended to be neutral regarding their susceptibility to COVID-19. The adoption of mass masking in public places in Hong Kong and the ability of the government in limiting the spread of disease may have led them to have this change in perception. The subjects’ positive belief that wearing a face mask could protect them from acquiring COVID-19 may have also contributed to this outcome.

Similar to the findings of having high levels of perceived seriousness of COVID-19, as reported during the early onset of the disease [42], the subjects in this study agreed that COVID-19 was a serious disease. The presence of reported death in Hong Kong and other countries is a promising evidence that makes the subjects believe that COVID-19 is a serious disease.

### Having actual inadequacy versus perceived inadequacy of face masks

During the initial three months in 2020, Hong Kong people experienced a period when the supply of face masks was extremely tense [43]. In late January 2020, pharmacies and stores in Hong Kong were running out of stock of face masks. Coupled with a number of factors, such as the announcement of shortage of face masks in hospitals in Mainland China and Hong Kong and the declaration of the COVID-19 outbreak as a global health emergency by the WHO, the supply gap of face masks has become even tenser than before. People in Hong Kong had to line up in extremely long queues for hours or even overnight simply to buy a box of face masks. Quotas were even set. Most people were disappointed when they knew that they could not obtain a quota. People also searched for face masks in online shops and outside Hong Kong but in vain. Panic buying was extremely common. In early March 2020, the supply was increased a bit. However, the price of a face mask remained relatively high, which was at least 10 times above the normal price.

This study was conducted in April 2020. In this period, people in Hong Kong were not in a desperate need for face masks. The supply of face masks became stable, and the prices were falling. The average cost for one face mask was HKD 0.5 (USD 0.064) before the COVID-19 pandemic, HKD 10 (USD 1.28) in January 2020, and HKD 5 (USD 0.64) in April 2020. The subjects, on average, owned 90 face masks. As a rough estimation, this quantity was sufficient for use in the next three months. Apparently, they should not have a problem of having inadequate face masks. The findings indicated that the subjects who reused and did not reuse face masks owned the same number of face masks. However, those who reused face masks perceived that they did not have adequate face masks. Instead of having an actual inadequacy of face masks, these subjects indicated a perceived inadequacy of face masks. Actual inadequacy represents an objective state of inadequacy, whereas perceived inadequacy represents a subjective interpretation of inadequacy.

The subjects in this study represented a population that is educated, mostly healthy, and is not living alone. They should have the ability to obtain face masks. The possible reason that makes them feel having inadequate face masks was discussed.

Recently, some Hong Kong people have exhibited a reduced level of trust in the government. The survey results on satisfaction with the performance of the Hong Kong government is reducing from 2.8 in April 2018 to 2.6 in April 2019 and further to 2.0 in April 2020 (minimum score = 1, maximum score = 5) [44]. The government’s reluctance to close entirely the land border with China, the shortages of face masks and other daily necessities in early 2020, and the slow implementation of some public health measures have intensified people’s level of mistrust [45].

In fact, the Hong Kong government has been relatively successful in protecting the population from COVID-19 and containing the pandemic, evidenced by having fewer confirmed cases and a flatter pandemic curve than other countries and regions where infections have soared [42]. Public health measures, such as border restrictions, social distancing, aggressive and extensive testing, rigorous contact tracing, quarantine, and isolation, that take place in Hong Kong likely play a part to limit the COVID-19 outbreak [12, 46, 47]. Hong Kong has also not been under full lockdown, which has been imposed in many countries [12, 48]. However, Hong Kong people mainly attribute this success to the optimum use of face masks by the general population.

With a reduced trust toward the government, some people in Hong Kong believe that they have to count on themselves. Wearing, stocking or even hoarding, and reusing face masks seem to be the few methods that they can do to protect themselves against COVID-19. In this study, the subjects agreed that wearing a face mask could protect them from acquiring COVID-19, and they tended to agree that they could protect themselves from acquiring the disease. The findings tentatively support the above analysis. However, further scientific investigation is necessary and recommended to confirm it.

### Limitations

This study has two limitations. First, this study recruited subjects through researchers’ networks. Two problems might have occurred. The first problem is related to the possibility of recruiting an unrepresentative sample. However, this problem should have been overcome greatly because the use of quota sampling ensured that the age and gender distribution of the sample resembled that of the population. Furthermore, the use of a large sample size counterbalanced the atypical results throughout the data collection process. The other problem is related to the possibility of collecting socially desirable answers from the subjects who knew the researchers. To reduce social desirability bias, subjects were reassured of data anonymity.

Second, this study used five questions to measure health beliefs. To be specific, it used one question to measure one aspect of health beliefs. The adequacy of the five questions is being challenged. Given that the five questions reported a high content validity index, their ability to measure the various aspects of health beliefs should not be a significant concern.

## Recommendations

This study paves the way for future studies to ascertain individuals’ practice in reusing face masks when the COVID-19 has been sustained for a year or even longer. Mostly, the health atmosphere will be different in two ways. First, individuals will be exposed to repeated waves of the disease. Up to May 2021, Hong Kong has experienced four waves of COVID-19 infection already. After exposure to repeated pandemic outbreaks, public fatigue in the use of face masks is likely to occur, and compliance will be reduced. A study on the Hong Kong population in a non-pandemic period presented a phenomenon of public fatigue [9]. Whether such phenomenon occurs in a pandemic period is uncertain. Second, an abundant and stable supply of face masks will be observed in the market. On top of the establishment of local production lines for face masks, social welfare bodies and volunteer groups have delivered free face masks to households with older adults and poor families. In this health atmosphere, individuals’ practice in using face masks is likely to be different.

Future research is advised to explore the possibility of recruiting subjects openly by accessing their telephone numbers from publicly available databases or randomly generated telephone numbers. In addition, future research is recommended to use an instrument with more questions to measure health beliefs to achieve a more comprehensive analysis and understanding. Moreover, future research is suggested to examine if factors other than health beliefs can influence individuals’ decision to reuse face masks and identify the underlying reasons behind this practice. Factors such as individuals’ knowledge and attitude on the health risk associated with the reuse of face masks are some of the examples. Other research topics, such as whether face masks can be reused and how face masks can be reused in a safe way, are also worthwhile of investigation. With a comprehensive understanding, the findings may provide pointers to the development of specific health promotion strategies on the proper use of face masks.

Health care professionals should instill effort to deliver message on the proper use of face masks to various populations through different platforms. Currently, health education strategies mainly focus on teaching the techniques on wearing and taking off a face mask and the moments when wearing face masks are indicated. An instruction on not recommending the reuse of face masks may be provided [8]. However, message on the health risk of reusing face masks is not provided. Thus, medical experts and practitioners should provide information on the undesirable consequences of reusing face masks and offer clarification on improper storage and decontamination of used face masks.

Community-based organizations or voluntary groups can contribute to support the affected population in this pandemic. Currently, some organizations have arranged delivery of complementary face masks to poor families and the older adults in various communities. Commonly, these activities are self-initiated, without coordination and lack of sustainability. A coordinated effort in establishing a platform to communicate information between donors and receiving parties and handling these services in a systematic manner can ensure that face masks can be evenly delivered to the affected population and boost the confidence of new parties to become involved.

The government has a responsibility to ensure a stable and adequate supply of face masks with reasonable price. Measures to support local production lines should be strengthened and continued. Concerned parties can also suggest a reference figure on the number of face masks for each individual to be kept at home. This figure can reassure individuals that they have adequate face masks and encourage them to avoid reusing them and keeping an excessive stock. The issue of distrust toward the government on individuals’ health practice is extremely complicated. However, the experience from other countries indicates that a timely response by the government when combating COVID-19 can lead to a desirable outcome [45].

## Conclusions

This study indicates that one-third of the adults in Hong Kong reuse face masks. They store and decontaminate their used face masks through various informal means. The practice of reusing a face mask is related to a health belief of having inadequate face masks. Future research is necessary to establish evidence and inform safe reuse. Concerted effort of health care professionals, community organizations, and the government will improve individuals’ practice in using face masks and alleviate their actual and perceived feeling of having inadequate face masks that will lead them to reuse.

## Data Availability

The datasets used during the current study are available from the corresponding author on reasonable request.

## Abbreviations

OR: odds ratio
CI: confidence interval
WHO: World Health Organization
